# Extracting Environmental Benefits from a New Canal in Nicaragua: Lessons from Panama

**DOI:** 10.1371/journal.pbio.1002208

**Published:** 2015-07-27

**Authors:** Richard Condit

**Affiliations:** Smithsonian Tropical Research Institute, Panama City, Republic of Panama

## Abstract

Biologists have raised objections to a new canal in Nicaragua, but in this Essay I argue that dire predictions of environmental catastrophe are exaggerated. I present an alternative view based on my research experience in Panama, where Canal operations foster forest conservation. Currently in Nicaragua, the rate of forest loss is so rapid that the canal cannot make it worse. Rather, I contend, adoption of international standards in canal construction could lead to net environmental and social benefits for the country.

## Abstract 2

Objections have been raised to a new canal in Nicaragua, but this Essay argues that those dire predictions are exaggerated; rather, international funding for the canal could be channeled into environmental protection while the project modernizes employment in a largely rural country.

## Introduction

**Fig 1:**
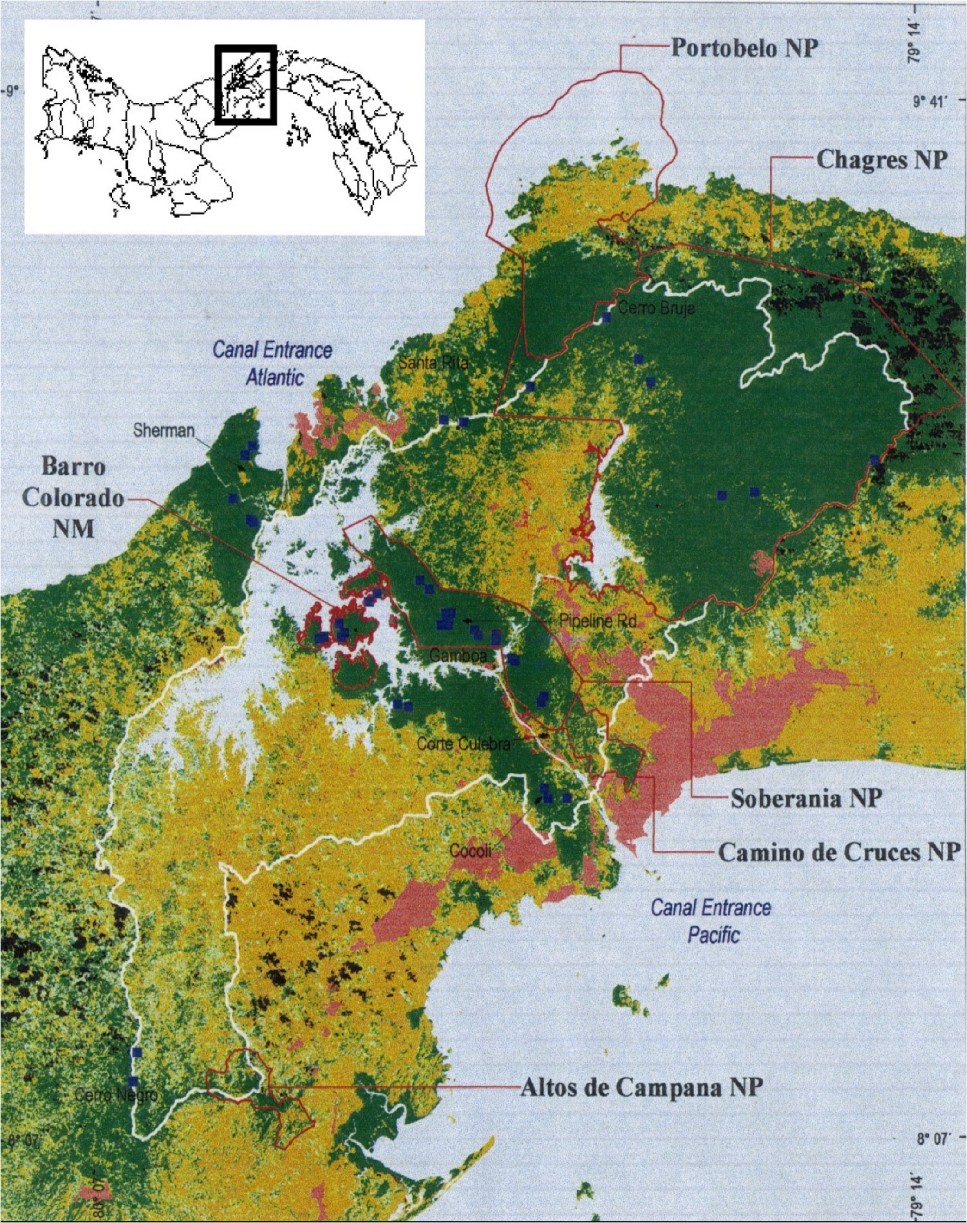
Forest around the Panama Canal. Forest cover (dark green) in the year 2000; little has changed since. The corridor extending from Caribbean to Pacific is the only place in Central America beyond the Panama-Colombia border where forest spans the isthmus. It is also among the best lowland tropical forest easily accessible to ecotourists anywhere. Without question, it would not be there had the US decided to put the Canal elsewhere.

Construction of a canal across Nicaragua will soon begin, completing a plan first proposed in the mid-19th century. The new canal will exceed the Panama Canal in size, cost US$40 billion, and provide infrastructure and jobs to a country that sorely needs both. Although there are claims that it will lead to environmental ruin (e.g. [1,2]), I argue, to the contrary, that a new canal might in fact advance environmental protection in Nicaragua. I base this assertion on 25 years of research in Panama, where I have studied the heavily forested Canal area and compared it with the rest of rural Panama, which is mostly farmland. There is no doubt to me that the decision to locate the Canal in Panama was the best environmental news the country ever received. From the beginning, the United States military and old US Canal Commission, ever concerned about security and water sources, restricted access to a 660 km^2^ corridor flanking the Canal, and the tall, diverse tropical forest there was fully protected. Much has changed in Panama since the early days of the 20th century, but government interest in protecting the forests of the Canal watershed has not (Fig 1). Now that Panama has taken over its Canal, the forests around it are intact and belong to Panama’s well-established system of national parks and nature monuments [3,4]. This forest would have been cleared last century without the protection of the US government, and deforestation would be well underway now without the current protection of the Panamanian government.

Panama is a useful precedent for examining resource requirements of a Nicaragua canal because the climate, natural habitats, and social context of the two countries are very similar. Both countries have wet climates naturally covered in tall, diverse forests, but humans have largely removed the forests to produce cattle and subsistence crops. Moreover, the new canal matches the existing Panama Canal in its main engineering features. In both designs, a canal is elevated 30 m above sea level across most of the isthmus; the elevated portion joins lakes that lie near each coast. In Panama, the two lakes are artificial, created by damming rivers, and in Nicaragua, the Caribbean lake will be created by damming the Punta Gorda River. On the Pacific side in Nicaragua, in contrast, it will be the natural Lake Nicaragua. Locks are needed to raise ships from the sea to the elevated lakes, and high rainfall allows the locks to be filled by existing stream flow, without recourse to pumping. The failure to recognize that a wet climate allows for passive filling is what undid the French Panama Canal Company’s effort in the 1890s. The French were certain a sea-level canal was the only option, and learned too late that dredging to sea level was beyond 19th century technology.

## Hydrology

Every time a ship crossing Panama is lowered to sea-level, a lock-full of water is dumped into the ocean (191,000 m^3^). Certainly inefficient, but the hydrological spreadsheet balances: 2–3 m of rain falling across the 3,000 km^2^ watershed is enough to fill the locks 14,000 times in most years [3,4]. But drought years test the limit. In 1935, a second dam was built on the Chagres above Lake Gatun to store more water, but even afterward, dry years (most recently, 1983 and 1997) forced the Panama Canal to bar passage to the largest ships. This is where forest protection enters the picture: forested land stores wet-season runoff more effectively than cleared land, and thus releases more during the dry season [3,4]. Based on this, the Panama Canal Authority, as well as ANAM (Panama's government authority responsible for National Parks), is keen on protecting the Atlantic-Pacific corridor of forest along the Canal and the spectacular forest upstream of Lago Alajuela in Chagres National Park (Fig 1).

Ships have gotten bigger in the last century, and the Panama Canal has finally embarked on a major expansion, the first since the original locks of 1910. The bigger locks will need more water, and after a century of discarding lock-fulls, a water-saving design is incorporated [5]. Ponds adjacent to the new locks will capture some of the discharged water each time, using gravity alone, and reduce water use by nearly a third. Nicaragua’s new locks are planned from the start with this water-saving trick, particularly important because the locks are larger, at 427 × 83 m [6]. Assuming one-third of the lock water is saved and 5,100 ships cross each year [7], approximately 3.3 km^3^ of water will be needed annually to run the locks. The water budget could be tight in dry years: given 2.2 m of precipitation falling over a 25,000 km^2^ watershed around the rift lakes, and 1.4 m lost to evapotranspiration, 20 km^3^ of water would flow into the lakes and canal. Currently, 9.6 km^3^ flow out of Lake Nicaragua into the San Juan River [8]; the difference between 20 and 9.6 is available to fill locks. The water budget is tight enough to demand careful management: the lesson in Panama is to protect forests in order to augment dry season flow and thus help assure canal operation through droughts.

## The State of the Environment: Central America

**Fig 2:**
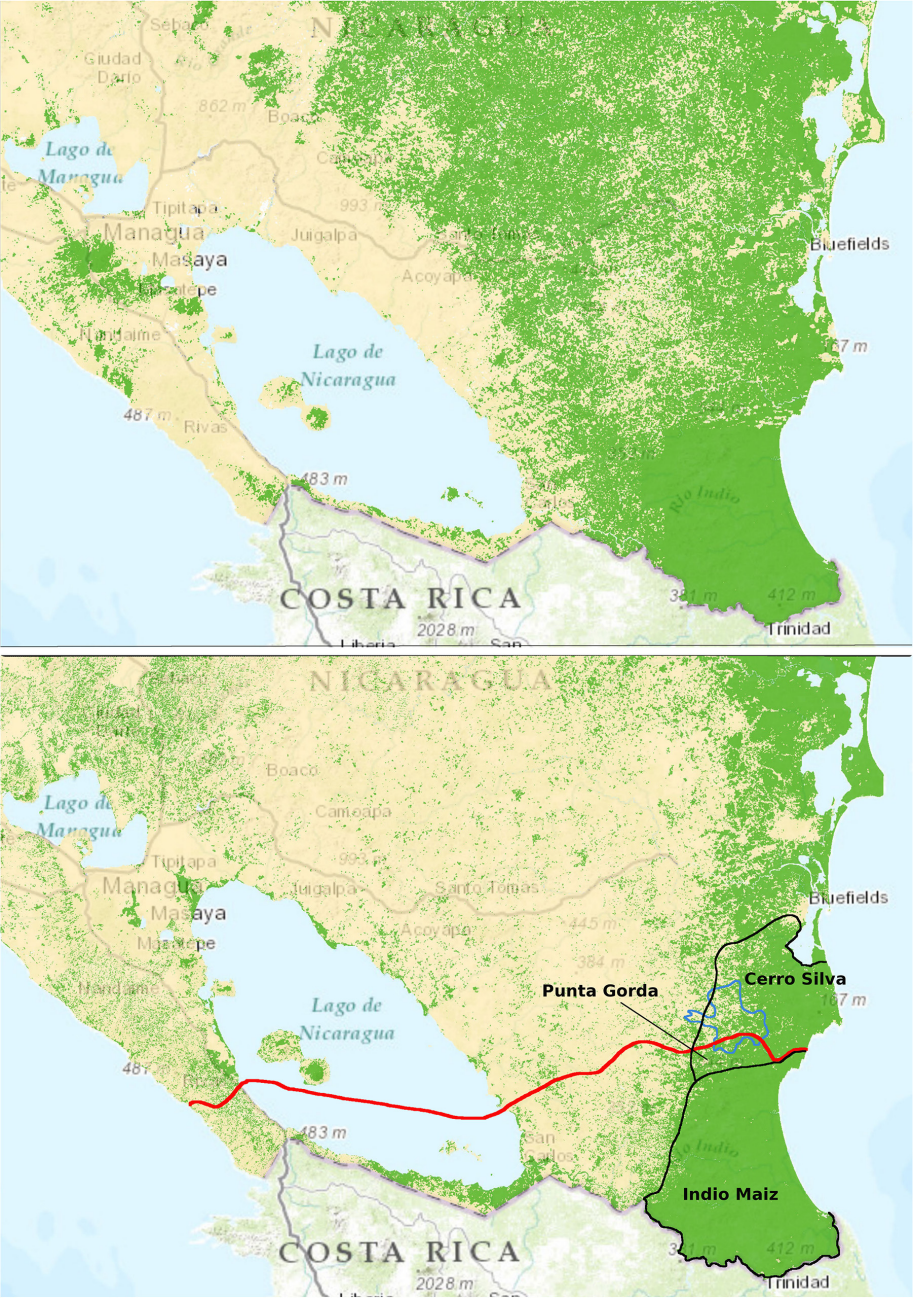
Route of the Nicaragua canal. The proposed route of a new canal in Nicaragua (red) against a backdrop of declining forest cover, 2000–2011 (dark green is forest; map from [9]). Three protected areas are outlined in black, and the to-be-dammed reservoir (Lago Atlanta) in blue. The canal’s entrance at the Caribbean and the new lake are in protected areas already being cleared for agriculture. Indeed, I encourage readers to check Google’s satellite images to see how much more forest has been cleared in those reserves since 2011, or use the interactive map showing global forest cover [11]. Indio Maiz remains largely intact up to 2014. *Image credit*: *Environmental Resources Management Group*, *Inc*.

The environmental impact of a new canal must be understood in the context of current environmental protection in Nicaragua. The central fact of the human environment in Central America is forest clearing; indeed, the land to be traversed by the new canal in Nicaragua is largely deforested. Most of the Pacific slope and the central part of the country is farmed and has been for decades, except for scattered forests on volcanic peaks. Deforestation east of Lake Nicaragua, however, is recent and happening quickly (Fig 2): 5,600 km^2^ around the central section of the proposed canal were cleared between 2000 and 2011 [9–11]. Nine thousand square kilometers of forest remain in that zone, and if recent rates of clearing continue, most could disappear in the next two decades. The remaining forests are mostly within 20 km of the Atlantic coast, a pattern typical throughout Central America, where the wet Caribbean slope still has low human occupation. But the growing Nicaraguan population is already pushing the agricultural frontier into protected areas near the Caribbean. Cerro Silva and Punta Gorda Reserves, between which the canal will pass, already have many clearings for subsistence agriculture. One last large block of unbroken Caribbean forest remains in Nicaragua, the 3,400 km^2^ Indio Maiz Reserve between the proposed canal and the Costa Rican border. Without improved management, protecting Indio Maiz and maintaining even 10%–20% of the original forest cover in large blocks, where the native biodiversity will persist intact, is not a sure bet over the next 25 years. It is an unfortunate fact that in both Nicaragua and Panama, as in many developing countries, needs for forest protection and management exceed institutional capacity.

Management of aquatic resources is similarly limited in Central America. Water quality is routinely poor, since sewage treatment is rare, and fisheries are unmanaged. Lake Nicaragua is a remarkable body of water, the largest lake in Central America, and was once home to freshwater sharks and stingrays, but the rays were fished heavily and are perhaps extinct [8,12]. In Panama, protection of the rivers and lakes feeding the Canal has helped maintain sport and commercial fisheries, and the dammed river and reservoirs are enormously popular for boating and bird-watching. There is an irony in this, though. No modern biologists saw what the Chagres was like before the Canal, and exotic fish, snails, and plants became plagues after dams and reservoirs were added. Nonetheless, biologists cherish the Chagres near Gamboa today as a lovely and diverse body of water surrounded by spectacular forest.

The situation in Nicaragua is, in most respects, parallel. A new canal will require continuous dredging across Lake Nicaragua to allow large ships to pass, and this will mean more silt, while those ships will probably raise the influx of potentially invasive exotics. Contrary to predictions of some environmentalists, however, shipping will require that the lake level be held within strict limits, and there is no reason to expect modern shipping to add to current levels of pollution [2]. Moreover, fears about deleterious impacts on the lake must be judged against its current status: as for forests, a new canal will not make the situation any worse.

## Economics

One aspect of the environmental impact of a new canal—resources consumed in building versus those saved later via reduced shipping distances—has been expressed in economic calculations. Indeed, the Panama venture was an enormous payoff to the world. Using present-day dollars as a metric, the Panama Canal cost US$8.5 billion to build, then saved the world shipping costs of US$2.1 billion per year during the 1920s and 1930s [13]. Ships paid 17% of those savings as tolls. Canal transits have doubled since then [14,15], so the Panama Canal is now saving the world US$4 billion each year. Resources saved (i.e., less fuel burned) from more efficient shipping are thus greatly exceeding the energy expended in building the Panama Canal. The Nicaragua canal could thus serve to reduce emissions of greenhouse gases and other pollutants in a similar manner, and some of the tolls could be invested in protecting the environment around the canal.

This depends, however, on the economics of the new canal. The US$40 billion price tag in Nicaragua is likely to be an underestimate, since an overrun of 50%–100% on such a large project would not be unexpected (the Panama Canal cost 2.1 times initial projections [13]). A cost of US$60–US$80 billion thus seems likely. Tonnage of world shipping has doubled since 1990 [16], and it would be wise to assume further increases in coming decades, so it is likely that a second Central American canal will serve a purpose. Will future savings in shipping costs beyond what the Panama Canal provides be sufficient to make the new canal a reasonable investment of resources? Will tolls pay for operation and protection of the canal’s environment? It is plausible but not certain. From a Nicaraguan perspective, though, the investment of foreign capital in the local economy would be an enormous boon. Labor opportunities associated with modernization, as the canal will provide, offer subsistence farmers economic advancement by leaving rural areas. With this comes the environmental benefit of reforestation on abandoned farmland and reduced pressure on existing forests.

## International Regulations

The investors from Hong Kong planning to build the Nicaragua canal agreed to meet international environmental standards, but it is not clear which standards. The International Finance Corporation (IFC) has published guidelines for reducing environmental impact that are the best standards available to international lenders [17]. If rigorously applied, IFC standards can help projects avoid unacceptable social and environmental damage, and in some scenarios even create a net positive environmental impact. For example, project funds can be used to protect habitat outside the footprint of development to compensate for what is destroyed on-site, and there are cases where IFC standards leveraged significant resources for conservation where little or none existed previously. In Mongolia and Madagascar, recent mining projects voluntarily adopted IFC standards, and both contributed management plans for protected areas adjacent to the mine sites [18,19]. In Panama, a foreign mining project is providing long-term financial support to protection of national parks around the impact site, reforestation on degraded land nearby, and a frog conservation center. The company’s 2015 contribution exceeds US$4 million, enormous in Panama’s environmental-protection budget, and will help conserve an area larger than the mine’s footprint (see [20]). While some may debate whether individual mitigation measures truly result in a “net positive impact,” it is a feasible goal, and the idea that international development includes environmental offsets is becoming a standard conservation tool [18]. If the Nicaragua canal were to be constructed in compliance with IFC standards, it would go a long way toward addressing concerns of the international community and enhancing environmental protection near the canal.

## A Practical Environmentalist’s Strategy

Any plan to help Nicaragua conserve its remaining forests should take into account two key points. First, given the financial hardships that the country and its citizens face, it is not reasonable to expect Nicaragua to balk at a project that would bring with it substantial foreign investment. Second, even without the canal, the current state of the environment in Nicaragua is so threatened that remaining forests are likely to disappear in a matter of decades. Maintaining the status quo is not a conservation plan. This suggests that a conservation strategy for Nicaragua’s forests—perhaps the only plausible one—is to design a canal project that also adds to the protection of significant natural habitat. In the case of a large canal, there is a clear synergy between the project and the need for the protection of natural habitats, because the canal administration will have considerable incentive to protect surrounding land, rivers, and lakes to ensure a water supply.

In order for the canal to live up to its potential as a conservation driver for Nicaragua, it should be constructed and operated in compliance with International Finance Corporation environmental and social standards. First, it is important that the Nicaraguan government make clear to the investors that it expects compliance with IFC standards. Second, an independent expert committee should be assembled to help review the recent environmental impact report [8] to ensure that the project delivers net economic, social, and ecological benefits to the country. From the perspective of biodiversity, at a minimum, mitigation measures should include long-term funding for the effective management of the Indio Maiz protected area, native forest regeneration in watersheds that are important to the functioning of the canal, and improved fisheries and pollution restrictions in Lake Nicaragua. Finally, as a condition for issuing a permit for the project, the investors should agree to ongoing and independent monitoring of the project and its compliance with environmental and social goals. If these conditions can be met, then the new canal may represent a tremendous opportunity to channel international investment in such a way that it generates significant economic and environmental benefits for a very poor country. This is where environmentalists should focus their efforts.
